# Evaluating the Carbon Emissions Efficiency of the Logistics Industry Based on a Super-SBM Model and the Malmquist Index from a Strong Transportation Strategy Perspective in China

**DOI:** 10.3390/ijerph17228459

**Published:** 2020-11-15

**Authors:** Xiaohong Jiang, Jianxiao Ma, Huizhe Zhu, Xiucheng Guo, Zhaoguo Huang

**Affiliations:** 1 College of Automobile and Traffic Engineering, Nanjing Forestry University, Longpan Road 159#, Nanjing 210037, China; majx@njfu.edu.cn (J.M.); teresazhz@163.com (H.Z.); 2 School of Transportation, Southeast University, Si Pai Lou 2#, Nanjing 210096, China; seuguo@163.com; 3 School of Civil Engineering, Lanzhou University of Technology, Lanzhou 730050, China; seuhuang369@hotmail.com

**Keywords:** logistics industry, carbon emissions, static efficiency, dynamic efficiency, super-slacks-based measuring model, Malmquist index

## Abstract

Carbon emissions from the logistics industry have been rising year after year. Correct handling of the relationship between economic development and environmental protection is of great significance to the implementation of green logistics, which is an important component of China’s strategy for strong transportation. This paper focuses on the evaluation of the carbon emissions efficiency of logistics industry from a new strong transportation strategy perspective. A super-efficiency slack-based measurement (Super-SBM) model and Malmquist index are combined to evaluate the static and dynamic carbon emissions efficiency of the logistics industry. The results indicate that compared with the SBM model, the Super-SBM model can more effectively measure the carbon emissions efficiency of the logistics industry. Pilot regions for the strong transportation strategy were divided into two categories, namely regions with slow carbon emission growth rates but high efficiency, and regions with high carbon emission growth rates but low efficiency. Some policy recommendations from the strong transportation strategy perspective were proposed to improve the carbon emissions efficiency of the logistics industry, especially for the second category of pilot regions. This study is expected to provide a basis for decision-making for efficient emissions reduction measures and policies, and to encourage the pilot regions to take the lead in achieving the goal of China’s strategy for transportation.

## 1. Introduction

China’s rapid economic growth has led to the depletion of resources and environmental degradation. Since 2005, China has accounted for approximately one-third of global carbon emissions and has become the world’s largest emitter of carbon dioxide [1]. China dominates global carbon emission trends and is under tremendous pressure to reduce emissions [2]. The logistics industry is a fundamental industry of the national economy. China’s total logistics revenue increased from 18.9 trillion US dollars (1 US dollar = 6.62 RMB) in 2010 to 41.1 trillion US dollars (1 US dollar = 6.89 RMB) in 2018. Meanwhile, logistics carbon emissions have also been increasing over the years, and this industry has the largest share of total energy consumption, the fastest growth, and the largest share of carbon emissions in China [3]. The China Energy Statistical Yearbook shows that the energy consumption of the logistics industry increased from 1315 tons in 1995 to 34.61 million tons in 2017, accounting for approximately 10 percent of China’s total energy consumption. Additionally, in recent years, the logistics industry has accounted for approximately 18 percent of the total carbon emissions for the entire country [4]. The main sources of carbon dioxide (CO_2_) generated in the logistics process are transportation and warehousing [5]. Statistics from Eurostat in 2005 show that the logistics industry accounts for approximately 5.5% of the global greenhouse gas emissions, of which 93% of the logistics CO_2_ emissions come from transport and 7% from storage [5]. The logistics performance is significantly related to environmental degradation [6]. According to an assessment report from the Intergovernmental Panel on Climate Change (IPCC), if current energy consumption patterns are maintained, the logistics industry will consume 80% more energy by 2030 than it does today [7]. China’s logistics industry is under tremendous pressure to save energy and reduce emissions [8]. Therefore, it is important to do research on the carbon emissions of China’s logistics industry in order to participate in global environmental governance and to fulfill carbon reduction commitments.

The Chinese government has performed a large amount of work towards achieving low-carbon targets for the logistics industry. The strategy of low-carbon logistics was incorporated into “China’s Thirteenth Five-Year Plan” [9]. This plan stated that low-carbon logistics technology and management methods should be promoted. In September 2019, the State Council issued a strategy for strong transportation, which is a new national development strategy for China [10]. China aims to increase its global competitiveness in the transport sector by constructing a strong transportation network [10]. The implementation of the strategy for a strong transportation network will inevitably bring changes and impacts to the logistics industry. An efficient and green logistics system is an important component in the construction of a strong transportation network. Correct handling of the relationship between economic development and environmental protection is very important in the implementation of green logistics [11]. By scientifically evaluating the static and dynamic carbon emissions efficiency of the logistics industry, a foundation could be laid for formulating policies for emissions reduction. Thirteen regions have been identified as the first batch of pilot regions for the strong transportation strategy. For this, regions in eastern, central, western, and southern China were selected. These pilot regions are representative and will play a leading role in the strong transportation strategy. Through cultivation and construction, the goal of building a strong transport system will be achieved in the pilot regions first, so that this process can be better promoted nationwide. In providing targeted policies and suggestions for each region, it is very important to encourage environmental protection and the long-term sustainable development of the logistics industry. Therefore, how to better assess the carbon emissions efficiency of the logistics industry and how to propose efficiency improvement measures from the perspective of a strategy for strong transportation are issues that need to be studied.

There have been many achievements in studies on the carbon emissions efficiency of the logistics industry [12]. Carbon emissions efficiency is an indicator that explains carbon emissions in a productivity framework. It is a measurement of the efficiency of CO_2_ emissions using the production frontier, i.e., the ratio between the actual output per unit of CO_2_ emissions and the optimal output of CO_2_ emissions. The combination of data envelopment analysis (DEA) and the Malmquist index is the most common method used to evaluate carbon emissions efficiency [13]. Conventional data envelopment analysis models, such as the Charnes–Cooper–Rhodes model (CCR) and the Banker–Charnes–Cooper model (BCC), have a wide range of applications in the area of static carbon emissions efficiency [4,11,14,15,16,17]. Several researches have conducted studies on the static and dynamic efficiency of logistics carbon emissions using DEA with the Malmquist index [4,17,18]. For example, Tian applied a CCR model to evaluate the emissions reduction efficiency of China’s provinces [14]. Yang applied the BCC model and Malmquist index to evaluate the logistics carbon emissions performance at the city level [4]. Lu evaluated the performance of green logistics in 112 countries using the environmental range adjusted measure (RAM)–DEA model [15]. Yang measured the logistics efficiency of regions along the Belt and Road area based on a three-stage DEA model [16]. Zhang applied the CCR model and Malmquist index to analyze the dynamic changes in logistics CO_2_ emissions performance [17]. Hui combined the BCC model and Malmquist index to measure the logistics CO_2_ emissions efficiency of provinces along the Silk Road economic belt [18].

However, the CCR and BCC models cannot deal with undesirable outputs [19]. Carbon emissions, as undesirable outputs, cannot be ignored in the logistics industry. The slack-based measurement (SBM) models proposed by Tone introduce slack variables into the objective function [20]. SBM models are gaining increasing attention from scholars for studying efficiency problems, including undesired outputs [13,21,22], and are also applied to achieve carbon emissions efficiency in logistics [23]. For example, Tang measured the transportation efficiency of the freight sector using the SBM model [22]. Mariano applied the SBM model to construct a logistics performance index ranking for a group of 104 countries [23]. Moreover, the efficiency values calculated by the SBM model range between 0 and 1. The super-efficiency slack-based measurement model (Super-SBM) proposed by Tone, is an improvement on the SBM model, allowing efficiency values greater than 1 to be used to further rank the SBM-efficient decision-making units (DMUs) [24]. The Super-SBM model has been applied in different areas, i.e., in industrial sectors [25], for low-carbon economy efficiency [26], in commercial banks [27], and for transportation carbon emissions efficiency [28,29], however little research has taken place in the context of logistics [30,31]. Wang computed the efficiency of port logistics companies base on the Super-SBM model [30], while Long combined Super-SBM model and the Malmquist–Luenberger index to evaluate the ecological logistics efficiency of regions along the Yangtze River economic belt [31].

From a research perspective, most of the existing studies have been conducted within countries [15], nationwide [6,15,23], in one province [14], in one city [4], in particular areas along the Silk Road economic belt [16], in the Belt and Road area [31], and in the Yangtze River economic belt [18]. However, there have not been any studies from the perspective of China’s strategy for strong transportation. The implementation of strong transportation strategies will inevitably bring changes and impacts to the logistics industry.

Taking the above issues into consideration, we aimed to propose a methodology to evaluate the static and dynamic carbon emissions efficiency of the logistics industry from a new perspective of China’s strategy for strong transportation. As carbon emissions is an undesirable output, the combination of Super-SBM and the Malmquist index is a suitable methodology. Fewer existing studies have adopted this methodology—only Long evaluated the logistics ecological efficiency based on the Super-SBM model and the Malmquist–Luenberger index [31]. However, he only studied regions along the Yangtze River economic belt, and did not analyze the reality from the perspective of China’s strong transportation network strategy. It is necessary to test the feasibility and superiority of this methodology. Therefore, the Super-SBM model was applied to measure the static carbon emissions efficiency of the logistics industry in the pilot regions from 2013 to 2017. To track changes in carbon emissions efficiency over time, we used the Malmquist index to calculate the dynamic carbon emission efficiency for two consecutive years of the research period. Based on the results, relevant improvement measures and policy implications were proposed to encourage the construction of a strong transportation network.

Relative to the existing research studies, we evaluated the carbon emissions efficiency of the logistics industry from the new perspective of a strategy for strong transportation. This is expected to provide a decision-making basis for formulating efficient emissions reduction measures in this context and to encourage the pilot regions to take the lead in achieving the goal of a strong transportation network. In addition, the applied methodology combining the Super-SBM model and the Malmquist index could give more precise efficiency values when evaluating the static and dynamic carbon emissions efficiency of the logistics industry. The Super-SBM model was applied to avoid two problems. One aim was to avoid the influence of slack variables on the carbon emissions efficiency measure. The other aim was to avoid situations in which the efficiency values measured by the conventional DEA model equal 1, which cannot be compared further. 

Following this introduction, Section 2 outlines the research methodology. Section 3 describes the results and discussion. Section 4 presents the conclusions.

## 2. Methodology

**Figure 1:**
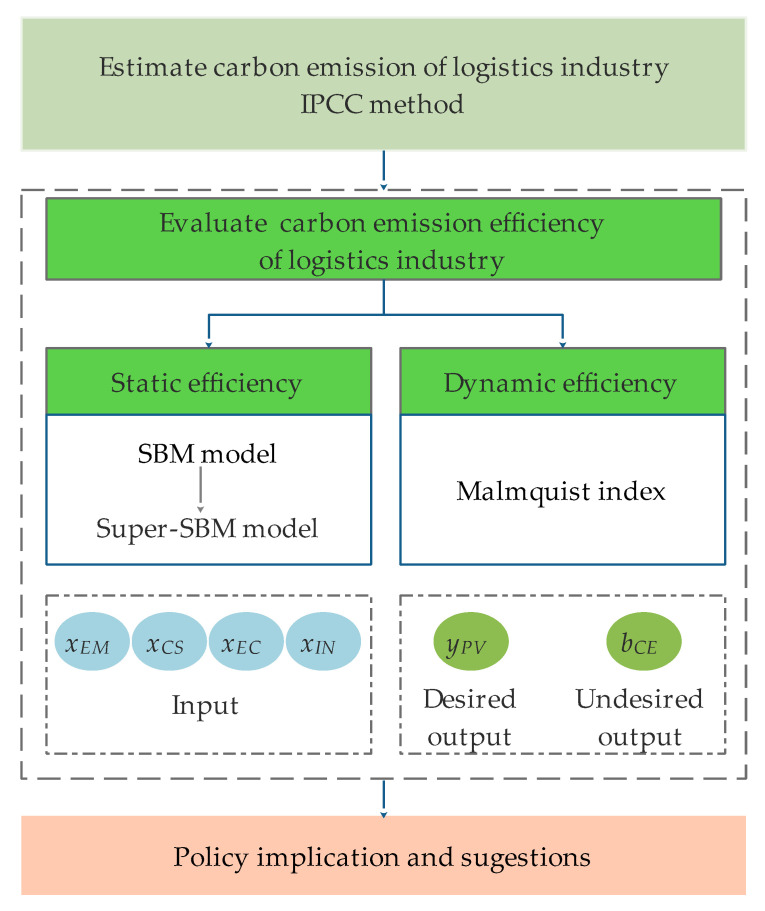
The research framework. IPCC: Intergovernmental Panel on Climate Change; SBM: slack-based measurement.

The method combining the Super-SBM model and the Malmquist index was applied to evaluate the static and dynamic carbon emissions efficiency of the logistics industry. The first batch of pilot regions for China’s strong transportation strategy was selected for this study. In order to conduct the evaluation, it was necessary to first estimate the carbon emissions of the logistics industry. The carbon emissions data for the logistics industry in China were not directly available. The IPCC method was applied here to estimate the carbon emissions of the logistics industry in the pilot regions from 2013 to 2017. The calculation method is described in Section 2.1. Then, the SBM model and Super-SBM model are introduced in Section 2.2 to evaluate the static carbon emissions of the logistics industry. By comparing the calculation results of SBM model and Super-SBM model, the superiority of the Super-SBM model is demonstrated. Furthermore, the Malmquist index described in Section 2.3 was applied to measure the dynamic carbon emissions of logistics industry. We chose employment (EM), capital stock (CS), energy consumption (EC), and infrastructure (IN) as the inputs, the production value (PV) as the desired output, and the carbon dioxide emissions (CE) as the undesired output. These variables and data sources are described in Section 2.4. Finally, based on the results, relevant policy recommendations were proposed. Figure 1 shows the research framework. 

### 2.1. Carbon Emissions Estimation

The carbon emissions estimation for the logistics industry uses the carbon emissions factor method proposed by IPCC [32], which has been widely used [4,12,14,15,16,23,31,33,34]. The emissions value is derived from the total consumption of logistics fuels multiplied by CO_2_ emission factors. The specific calculation is shown in Equation (1):

(1) $$C=\sum_{i=1}^{5}E_{i}\times NCV_{i}\times CEF_{i}\times COF_{i}\times \frac{44}{12}$$

where *C* is the estimated value of the CO_2_ emissions, *E_i_* represents the consumption of each type of energy, *NCV_i_* signifies the average low calorific value, *CEF_i_* denotes the carbon emissions coefficient, *COF_i_* is the carbon oxidation factor, and 12 and 44 represent the molecular weights of carbon and carbon dioxide, respectively. According to the statistical data from the China Energy Statistical Yearbook and the general rule of comprehensive energy consumption [35], the seven types of energy that are commonly used in the logistics industry were defined, which are listed in Table 1.

### 2.2. Data Envelopment Analysis Method

#### 2.2.1. Slack-Based Measurement Model

The DEA is a non-parametric method used to evaluate the relative efficiencies of a set of comparable decision-making units (DMUs) with several inputs and outputs. The CCR and BCC model the DMUs indiscriminately on the same frontier and do not account for the impact of the relaxation variables on the efficiency measurements [19]. When the CCR and BCC models are applied to solve the undesired output, they must be converted into input indicators or processed using a distance function or curve measurement assessment. However, this conversion undermines the realism of the data itself and leads to a reduction in the realism of the efficiency measurements [36]. The non-linear conversion method in the data conversion function does not satisfy the convexity requirements of the conversion process. In 2001, Tone introduced relaxation variables into the objective function by developing a non-angular and non-radial slack-based measurement (SBM) model [20]. The SBM model addresses the problem of input relaxation and is a good solution to the problem of energy efficiency evaluation where undesired outputs are included. 

Under the framework of total factor productivity, the static carbon emissions efficiency index of the logistics industry (SLCEI) was measured on the basis of the carbon emissions and the influence of the undesired output on the carbon emissions efficiency. A slack-based measurement model (SBM) with undesired outputs was applied to evaluate the carbon emissions efficiency of the logistics industry and to avoid two problems. One aim was to avoid the influence of slack variables on the carbon emissions efficiency measure. The other was to avoid the situations in which the efficiency values measured by the conventional DEA equal 1, which cannot be compared [13].

We assume that there are *n* DMUs, i.e., the twelve pilot regions. Each DMU has *m* inputs, *s_1_* desired outputs, and *s_2_* undesired outputs, which are represented by the vectors $x\in R^{m}$, $y\in R^{s_{1}}$, $b\in R^{s_{2}}$, respectively. We chose employment (EM), capital stock (CS), energy consumption (EC), and infrastructure input (IN) as inputs, the production value (PV) as the desired output, and the carbon dioxide emissions (CE) as the undesired output. Here, we define the matrix as $X=\left[x_{EM},\;x_{CS},x_{EC},\;\;\;x_{IN}\right]\in R^{4\times 12}$, $Y=\left[y_{PV}^{}\right]\in R^{1\times 12}$. Assuming *X > 0, Y > 0, B > 0*, the set of production possibilities, *P*, is defined as in Equation (2):

(2) $$P(x,y,b)=\left\{\left(X,\;\;\;Y,\;\;\;B\right)|x\geq X\lambda ,\;\;y\leq Y\lambda ,\;b\geq B\lambda \;,\;\;\sum_{i=1}^{n}\lambda =1,\;\lambda \geq 0\;\right\}$$

where *λ* is the weight of the efficiency measure. If the sum of the weights is 1, this represents a variable scale reward. If the sum of the weights is not 1, it is a constant scale reward. According to the research by Tone [20], the SLCEI under the SBM model is defined as in Equation (3):

(3) $${\mathrm{SLCEI}}_{\mathrm{SBM}}=min\frac{1-\frac{1}{4}\left(\frac{s_{EM}^{-}}{x_{EM}}+\frac{s_{CS}^{-}}{x_{CS}}+\frac{s_{EC}^{-}}{x_{EC}}+\frac{s_{IN}^{-}}{x_{IN}}\right)}{1+\frac{1}{2}\left(\frac{s_{PV}^{+}}{y_{{}_{PV}}^{}}+\frac{s_{CE}^{-}}{b_{{}_{CE}}^{}}\right)}\;s.t.\;\left\{ \begin{array}{l} \;x_{EM}=\sum_{j=1}^{12}\lambda_{j}x_{EMj}+s_{EM}^{-}\\ x_{CS}=\sum_{j=1}^{12}\lambda_{j}x_{CSj}+s_{CS}^{-}\\ x_{EC}=\sum_{j=1}^{12}\lambda_{j}x_{ECj}+s_{EC}^{-}\\ x_{IN}=\sum_{j=1}^{12}\lambda_{j}x_{INj}+s_{IN}^{-}\\ \;y_{PV}^{}=\sum_{j=1}^{12}\lambda_{j}y_{PVj}^{}-s_{PV}^{-}\\ b_{CE}^{}=\sum_{j=1}^{12}\lambda_{j}b_{CEj}^{}+s_{CE}^{-}\\ s_{EM}^{-},\;\;\;s_{CS}^{-},\;\;\;s_{EC}^{-},\;\;\;s_{IN}^{-},s_{PV}^{+},s_{CE}^{-},\;\;\;\;\;\lambda_{j}\geq 0,\;\;\;j=1,2,\ldots ,12\\ \sum_{j=1}^{12}\lambda_{j}=1 \end{array}\right.$$

where $s_{EM}^{-}$, $s_{CS}^{-}$, $s_{EC}^{-}$, $s_{IN}^{-}$, $s_{PV}^{+}$, and $s_{CE}^{-}$ denote slack in the inputs, desired outputs, and undesired output, respectively; $s_{EM}^{-}$, $s_{CS}^{-}$, $s_{EC}^{-}$, and $s_{IN}^{-}$ indicate that the actual input resource is greater than the frontier investment; $s_{PV}^{+}$ indicates that the desired output produced in the realistic operation is less than the frontier desirable output; $s_{CE}^{-}$ means that the actual undesirable output level is greater than the leading edge of the undesirable output level. The range of values for the SLCEI is [0,1]. When SLCEI = 1, the production unit is fully efficient, there are no excesses of inputs and undesired outputs, and there is no shortage of desired outputs. When SLCEI < 1, there is an efficiency loss in the production unit, and the efficiency value can be improved by optimizing the quantities of inputs and outputs.

#### 2.2.2. Super-Efficiency Slack-Based Measurement Model 

The efficiency values calculated by the SBM model range between 0 and 1. In most cases, there may be multiple SBM-efficient DMUs, i.e., their efficiency values are equal to 1 [13]. In this case, the model cannot further rank the SBM-efficient DMUs. In 2002, Tone developed the super-efficiency slack-based measurement model (Super-SBM), which is an improvement on the SBM model, allowing efficiency values greater than 1 to be used to further rank the SBM-efficient DMUs [24]. It is assumed that the production possibilities set *P’* is defined by excluding (*x*_0_, *y*_0_, *b*_0_) from (*X, Y, B*), as in Equation (4). The subset $\bar{P}$ of $P^{\prime }$ can be defined as Equation (5):

(4) $$P^{\prime }(x_{0},y_{0},b_{0})=\left\{\left(\bar{x},\;\;\bar{y},\;\;\bar{b}\right)|\bar{x}\geq X\lambda ,\;\;\bar{y}\leq Y\lambda ,\;\;\bar{b}\geq B\lambda ,\bar{y}\geq 0\lambda \geq 0\right\}$$

(5) $$\bar{P}(x_{0},y_{0},b_{0})=P^{\prime }(x_{0},y_{0},b_{0})\cap \left\{\bar{x}\geq x_{0}\;\;,\;\;\bar{y}\leq y_{0},\bar{b}\geq b_{0}\right\}$$

The subset is not empty when the input–output is larger than 0. In accordance with the Super-SBM model developed by Tone [24], the SLCEI under the Super-SBM model is defined as Equation (6):

(6) $${\mathrm{SLCEI}}_{S\text{-}\mathrm{SBM}}=min\frac{\frac{1}{4}\left(\frac{\bar{x}}{x_{EM}}+\frac{\bar{x}}{x_{CS}}+\frac{\bar{x}}{x_{EC}}+\frac{\bar{x}}{x_{IN}}\right)}{\frac{1}{2}\left(\frac{\bar{y}}{y_{PV}}+\frac{\bar{b}}{b_{CE}}\right)}\;s.t.\;\left\{ \begin{array}{l} \bar{x}\geq \sum_{j=1}^{12}\lambda_{j}x_{EMj}+\sum_{j=1}^{12}\lambda_{j}x_{CSj}+\sum_{j=1}^{12}\lambda_{j}x_{ECj}+\sum_{j=1}^{12}\lambda_{j}x_{INj}\\ \bar{y}\leq \sum_{j=1}^{12}\lambda_{j}y_{PVj}^{}\\ \bar{b}\geq \sum_{j=1}^{12}\lambda_{j}b_{CEj}^{}\\ \bar{x}\geq x_{EM},\bar{x}\geq x_{CS},\bar{x}\geq x_{EC},\bar{x}\geq x_{IN}\\ \bar{y}\leq y_{PV}^{}\\ \;\;\bar{b}\geq b_{CE}^{},\;\\ \lambda_{j}\geq 0,j=1,2,\ldots ,12\\ \sum_{j=1}^{12}\lambda_{j}=1 \end{array}\right.$$

This SLCEI was decomposed into the pure technical efficiency (PTE) and scale efficiency (SE). Mathematically, SLCEI=PTE×SE. The PTE represents the ratio of the distance between the actual level of output and the level of returns to scale, which reflects the use of technology. SE represents the change in the payoff to scale of the DMU. 

### 2.3. Malmquist Index

The Malmquist index is widely used to study the dynamics of production efficiency. It was first introduced by Sten Malmquist in 1953 to study the evolution of consumption over time [13]. Fare extended the Malmquist index by decomposing productivity growth into technology changes and efficiency changes using non-parametric programming methods [37]. We proposed a dynamic carbon emissions efficiency index for the logistics industry (DLCEI) to evaluate the changes in carbon emissions from 2013 to 2017. According to the research on the Malmquist index proposed by Fare [37], the DLCEI is defined as shown in Equation (7):

(7) $${\mathrm{DLCEI}}_{j}^{t,t+1}={\left[\frac{D_{j}^{t}\left(x_{EM}^{t+1},x_{CS}^{t+1},x_{EC}^{t+1},x_{IN}^{t+1},\;y_{PV}^{t+1},\;b_{CE}^{t+1}\right)}{D_{j}^{t}\left(x_{EM}^{t},x_{CS}^{t},x_{EC}^{t},x_{IN}^{t},\;y_{PV}^{t},\;b_{CE}^{t}\right)}\times \frac{D_{j}^{t+1}\left(x_{EM}^{t+1},x_{CS}^{t+1},x_{EC}^{t+1},x_{IN}^{t+1},\;y_{PV}^{t+1},\;b_{CE}^{t+1}\right)}{D_{j}^{t+1}\left(x_{EM}^{t},x_{CS}^{t},x_{EC}^{t},x_{IN}^{t},\;y_{PV}^{t},\;b_{CE}^{t}\right)}\right]}^{\frac{1}{2}}$$

where DLCEI is defined as the Malmquist index, which measures the dynamic change of the *j*th DMU in the Malmquist index between period *t* and period *t* + 1; $D_{j}^{t}\left(x_{EM}^{t},x_{CS}^{t},x_{EC}^{t},x_{IN}^{t},\;y_{PV}^{t},\;b_{CE}^{t}\right)$ and $D_{j}^{t+1}\left(x_{EM}^{t},x_{CS}^{t},x_{EC}^{t},x_{IN}^{t},\;y_{PV}^{t},\;b_{CE}^{t}\right)$ are the distance function of $\left(x_{EM}^{t},x_{CS}^{t},x_{EC}^{t},x_{IN}^{t},\;y_{PV}^{t},\;b_{CE}^{t}\right)$ in period *t* and period *t* + 1, respectively. If DLCEI > 1, the Malmquist index is trending upward and increasing in efficiency from period t to period *t* + 1. If DLCEI < 1, the Malmquist index is trending downward, and the efficiency decreases. If DLCEI = 1, the Malmquist index remains unchanged from period *t* to period *t* + 1, and the efficiency remains unchanged.

This Malmquist index can be decomposed into two components, namely changes in technology (TCH) and changes in efficiency (ECH), as in Equations (8) and (9) [37]. Mathematically, DLCEI=TCH×ECH.

(8) $${\mathrm{TCH}}_{j}^{t,t+1}={\left[\frac{D_{j}^{t}\left(x_{EM}^{t+1},x_{CS}^{t+1},x_{EC}^{t+1},x_{IN}^{t+1},\;y_{PV}^{t+1},\;b_{CE}^{t+1}\right)}{D_{j}^{t+1}\left(x_{EM}^{t+1},x_{CS}^{t+1},x_{EC}^{t+1},x_{IN}^{t+1},\;y_{PV}^{t+1},\;b_{CE}^{t+1}\right)}\times \frac{D_{j}^{t}\left(x_{EM}^{t},x_{CS}^{t},x_{EC}^{t},x_{IN}^{t},\;y_{PV}^{t},\;b_{CE}^{t}\right)}{D_{j}^{t+1}\left(x_{EM}^{t},x_{CS}^{t},x_{EC}^{t},x_{IN}^{t},\;y_{PV}^{t},\;b_{CE}^{t}\right)}\right]}^{\frac{1}{2}}$$

(9) $${\mathrm{ECH}}_{j}^{t,t+1}=\frac{D_{j}^{t}\left(x_{EM}^{t+1},x_{CS}^{t+1},x_{EC}^{t+1},x_{IN}^{t+1},\;y_{PV}^{t+1},\;b_{CE}^{t+1}\right)}{D_{j}^{t}\left(x_{EM}^{t},x_{CS}^{t},x_{EC}^{t},x_{IN}^{t},\;y_{PV}^{t},\;b_{CE}^{t}\right)}$$

The TCH reflects the extent to which the efficiency frontier has been moved and is used to measure the application of new technologies and products by the DMU. ECH is the degree of change in the technical efficiency of the DMU from period *t* to period *t* + 1. When ECH > 1, the technical efficiency has improved, i.e., the relevant management approach is correct. When ECH < 1, a decrease in the technical efficiency is indicated, i.e., the relevant management approach is inappropriate.

### 2.4. Variables and Data Description

The first batch of pilot regions were taken as an example. The Hebei Xiongan New Area in this group of pilot regions is still under construction, and no relevant data are available. In Shenzhen, where the data are difficult to obtain, Guangzhou province is used as a substitute. Therefore, twelve provinces and cities were selected as research objects, namely Liaoning Province, Jiangsu Province, Zhejiang Province, Shandong Province, Henan Province, Hubei Province, Hunan Province, Guangxi Zhuang Autonomous Region, Chongqing Municipality, Guizhou Province, Xinjiang Uygur Autonomous Region, and Guangdong Province.

Because the logistics industry is a new industry, in China’s “National Economy Industry Classification” of 20 categories, the logistics industry was not listed separately. There is a lack of statistical data on the logistics industry in China. The vast majority of existing studies are based on data from transportation, warehousing, and postal services as a substitute for logistics [4,14,16,31]. These three industries account for approximately 80% of the overall logistics industry [31]. We adopted this alternative approach. 

We chose employment, capital stock, energy consumption, and infrastructure as the inputs, the production value as the desired output, and the carbon emissions as the undesired output [14,16,38]. The data for these indicators were obtained from the China Energy Statistical Yearbook (2013–2017) and the China City Statistical Yearbook (2013–2017). The China Energy Statistics Yearbook contains energy statistics data collected and organized by the Energy Department of the National Bureau of Statistics. It is an authoritative source that comprehensively reflects China’s energy construction, production, consumption, and supply and demand balance. The China City Statistical Yearbook is an annual publication that comprehensively reflects the economic and social development of Chinese cities, and is sponsored by the Department of Urban Socioeconomic Survey of the National Bureau of Statistics of China. Table 2 gives a description of the data sources for each indicator.

In the China Energy Statistical Yearbook (2013–2017), the energy consumption data for the logistics industry in Zhejiang province is missing due to the absence of regional data and for other reasons. Therefore, we can only calculate the energy consumption for Zhejiang province based on the energy conversion ratio.

## 3. Results and Discussions

### 3.1. Carbon Emissions of the Logistics Industry

**Figure 2:**
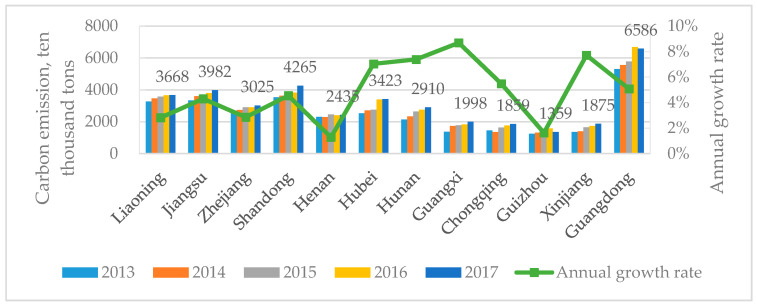
Carbon emissions (10^4^ tons) for the logistics industry in the pilot regions from 2013 to 2017.

**Figure 3:**
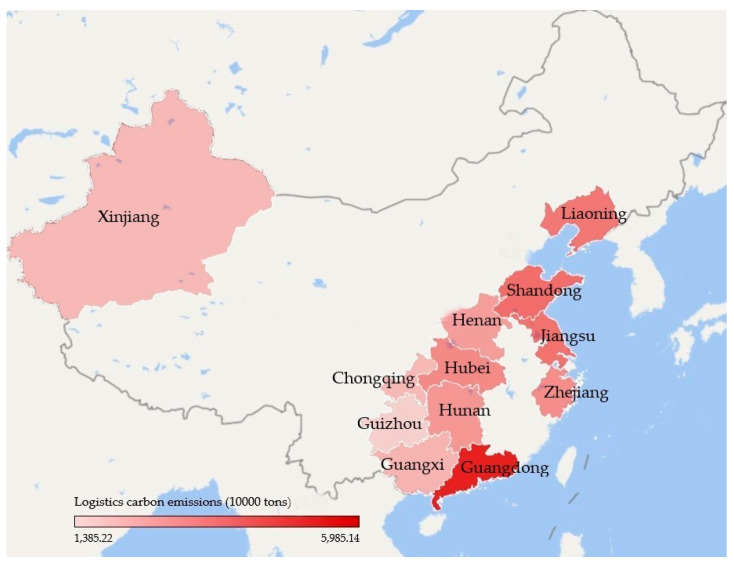
Average annual carbon emissions (10^4^ tons) for the logistics industry in the pilot regions.

The carbon emissions of the logistics industry in the twelve pilot regions were estimated using Equation (1). The results in Figure 2 show that the carbon emissions increased in the majority of the pilot regions throughout the research period. Figure 2 represents the total carbon emissions for each pilot region in 2017. Guangdong, Shandong, and Jiangsu are the top three largest carbon emitters, while Guizhou, Xinjiang, and Chongqing have the lowest carbon emissions. The line in Figure 2 represents the average annual growth rate for the carbon emissions (AGRCE). We found that the growth rate was usually faster in low-emission regions, namely Xinjiang, Chongqing, Guangxi, Hunan, and Hubei. However, Guizhou has very low carbon emissions and a slow growth rate. By plotting the average annual carbon emissions of each pilot region on a map (Figure 3), the location of each pilot region and the differences in carbon emissions can be clearly seen; from the west to the east, carbon emissions are increasing.

### 3.2. Carbon Emissions Efficiency of the Logistics Industry

#### 3.2.1. Static Carbon Emissions Efficiency of the Logistics Industry 

Using the panel data for the pilot regions from 2013 to 2017, the values for the static carbon emissions efficiency index for the logistics industry (SLCEI) under the SBM and Super-SBM models were calculated using Equations (3) and (6), respectively. The results are shown in Table 3.

By comparing the results for the SBM model and Super-SBM model, it can be seen that the values are basically consistent. There are seven SBM-efficient DMUs (i.e., DMUs for which the efficiency values are equal to 1 simultaneously), which are marked in bold in Table 3. By applying the Super-SBM model, the efficiency values for these seven SBM-efficient DMUs were further calculated and compared. This proved that Super-SBM could provide more accurate relative efficiency values when comparing all DMUs. The following analysis focuses on the SLCEI results obtained from the Super-SBM model. 

The average SLCEI values for the 12 pilot regions varied considerably, from a minimum of 0.306 to a maximum of 0.977. The 12 pilot regions were ranked based on the average annual SLCEI values. Jiangsu had the highest SLCEI value, which was basically 1 per year. Jiangsu is located in the Yangtze River Delta region, the fastest growing region in terms of China’s logistics industry. This SLCEI indicated that Jiangsu had basically formed a more mature logistics system and was able to control the CO_2_ emissions more reasonably [31]. Shandong had the second highest average SLCEI of 0.85. The excellent coastline and port conditions in Shandong have provided a good base to develop international trade and shipping logistics [23]. 

Guizhou, Henan, Liaoning, Zhejiang, Hunan, and Guangdong were in the second tier of the SLCEI, with average values of between 0.6 and 0.8. Guizhou had very low carbon emissions and its average SLCEI was 0.8 because of the smaller proportion of industry [14]. Henan and Hunan belong to the central part of China, having a well-connected transport network. Liaoning is an important transportation hub for the three eastern provinces, with relatively perfect transportation infrastructure [14]. Liaoning’s SLCEI has been increasing in recent years, indicating that the development of green and low-carbon logistics has been going well. Zhejiang is located in the Yangtze River Delta region, with an average SLCEI of 0.7. Guangdong was the largest carbon emitter because of its dense population and its higher proportion of industry, and its average SLCEI was 0.62. The SLCEI values of these pilot regions were less than 1, however it is still necessary to continue to develop low-carbon logistics networks and to undertake a series of measures to improve the carbon emissions efficiency. 

The lowest SLCEI scores were in Xinjiang, Chongqing, Hubei, and Guangxi provinces, with average values below 0.4. Notably, Xinjiang province had the lowest SLCEI, with an average value of 0.306. Guangxi, Chongqing, and Xinjiang provinces belong to the economically underdeveloped western region. Due to the “Western Development” strategy, the transportation network and the logistics industry of Xinjiang are developing rapidly. However, the large-scale financial investment in the logistics industry has not been immediately translated into increased production capacity [18]. Thus, the carbon emissions efficiency of the logistics industry in this area has fluctuated across a lower range. 

**Figure 4:**
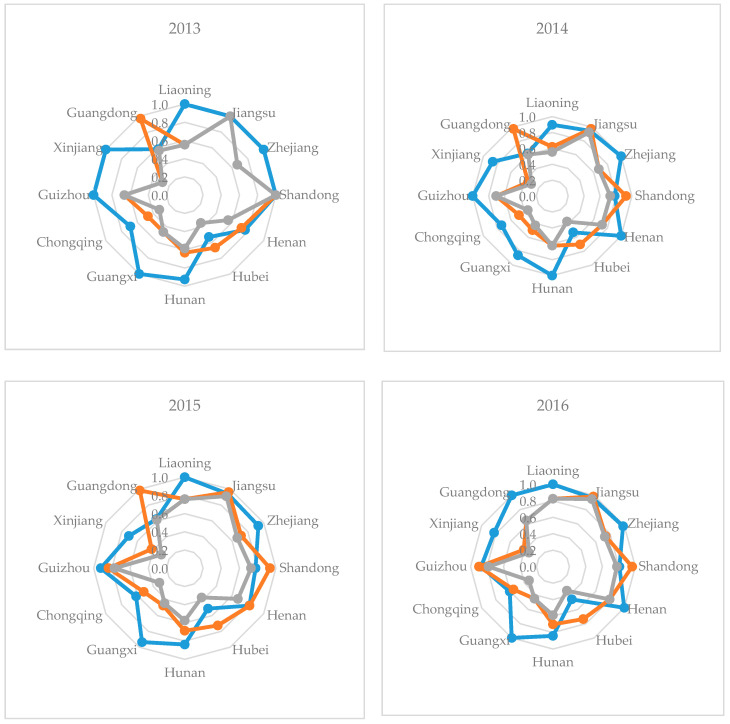

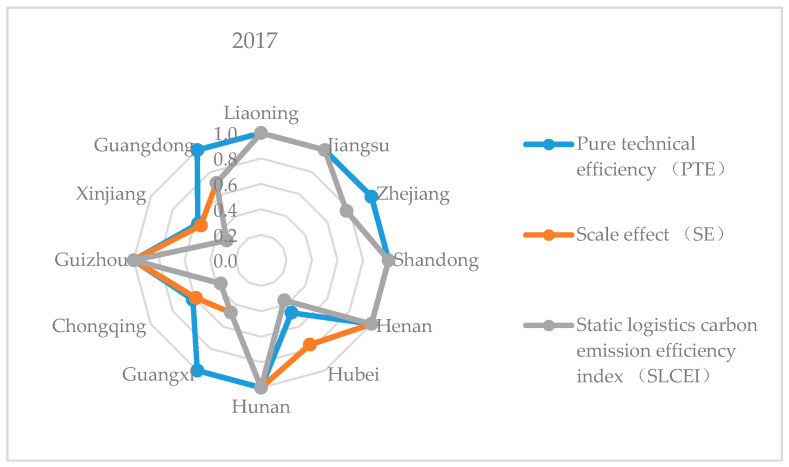
Static carbon emissions efficiency index for the logistics industry (SLCEI) along with the pure technical efficiency (PTE) and scale efficiency (SE) values in the pilot regions.

Furthermore, the SLCEI can be decomposed into the pure technical efficiency (PTE) and scale efficiency (SE), and the results for each pilot region are shown in Figure 4. The increases in the pure technical efficiency were basically greater than the increases in the scale efficiency. The differences among the twelve pilot regions in SLCEI were mainly due to the differences in the scale efficiency. Only Hubei and Shandong were different, whereby their PE values were smaller than the SE values. These two regions need to further improve their pure technical efficiency in order to achieve greater carbon emissions efficiency. The lower regions in the SLCEI rankings had very low PTE and SE values, such as Xinjiang, with an SE value of only 0.282 in 2013. Between 2013 and 2017, the PTE value decreased from 1 to 0.572 and the SE value increased from 0.282 to 0.545. The average annual SLCEI for Chongqing was 0.339. The PTE value was approximately 0.6 and the SE value was approximately 0.3. 

#### 3.2.2. Dynamic Carbon Emissions Efficiency of the Logistics Industry

Equation (7) was applied to calculate the Malmquist index and obtain the dynamic carbon emissions efficiency index for the logistics industry (DLCEI) values for each pilot region from 2013 to 2017. The DLCEI results are shown in Table 4. 

From Table 4, it can be seen that the differences in the DLCEI values among the twelve pilot regions were very small; almost all of them were greater than or equal to 1, with only Hubei being less than 1. This finding indicates steady increases in the overall carbon emissions efficiency for all elements of the logistics industry. The DLCEI values for Liaoning, Jiangsu, Zhejiang, Shandong, Henan, Hunan, and Guizhou provinces were all greater than 1, indicating that the carbon emissions efficiency of the logistics industry in the above pilot regions is increasing. Additionally, the above regions have better technical support in terms of carbon emissions pollution control. The logistics industry moved toward the use of contemporary technology from 2013 to 2017, as reflected by the increased efficiency in terms of carbon emissions performance.

The DLCEI values for Chongqing and Xinjiang were 1.033 and 1.038, respectively. Combined with the SLCEI results calculated in the previous section, it can be seen that although the logistics industry in these two regions was less efficient during 2013–2017, the growth rate has been faster in recent years. In recent years, the migration of Chinese industries from the east to the central and western regions has offered a rare opportunity for green logistics development in the central and western regions. 

Hubei province has the third lowest SLCEI value and the lowest DLCEI value, with an annual average value of 0.998. These findings indicate that the carbon emissions efficiency of the logistics industry in Hubei province is at a relatively low level and has not improved in recent years.

**Figure 5:**
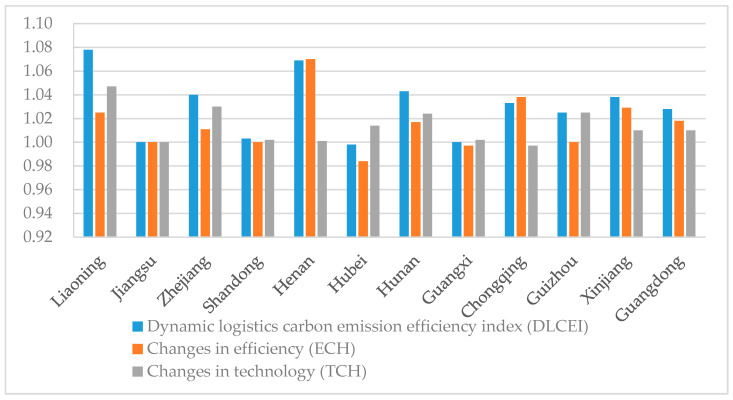
Annual average dynamic carbon emissions efficiency of the logistics industry (DLCEI) and decomposition of each pilot region into TCH and ECH values.

Furthermore, the DLCEI was decomposed into TCH and ECH values for each pilot region during 2013 and 2017, which were calculated using Equations (8) and (9) and are shown in Figure 5. Jiangsu’s TCH and ECH values were both 1, indicating that Jiangsu is advancing in terms of technology changes and efficiency improvement in parallel. Seven regions had ECH values that were lower than their TCH values, namely Liaoning, Guizhou, Guangxi, Hunan, Hubei, Shandong, and Zhejiang. This means that these seven regions’ changes in efficiency were lower than the changes in technology. Four regions had ECH values larger than TCH values, namely Shandong, Xinjiang, Chongqing, and Henan. This means that these four regions’ changes in efficiency were larger than the changes in technology.

### 3.3. Discussions

We first discussed logistics carbon emissions, the static carbon emissions efficiency index (SLCEI), and the dynamic carbon emissions efficiency index (DLCEI) separately, then plotted all three on a single graph for discussion. Finally, some suggestions and recommendations for pilot regions were proposed. Based on the above results for logistics carbon emissions, SLCEI, and DLCEI, we found the following insights.

The logistics carbon emissions for the majority of pilot regions increased throughout the research period. The average annual growth rates for carbon emissions were generally faster in low-emission regions, namely Xinjiang, Chongqing, Guangxi, Hunan, and Hubei.

There were seven SBM-efficient DMUs, i.e., with efficiency values equal to 1. By applying the Super-SBM model, the efficiency values for these seven SBM-efficient DMUs were further calculated and compared. This proved that Super-SBM could provide more accurate relative efficiency values when comparing all DMUs.

The average SLCEI values for the twelve pilot regions varied considerably, from a minimum of 0.306 to a maximum of 0.977, showing a general trend of growth over this research period. The pure technical efficiency (PTE) was larger than the scale efficiency (SE) in the majority of pilot regions. This indicated that the scale efficiency must first be improved. The lowest carbon emissions efficiency values were in Xinjiang, Chongqing, and Hubei provinces, with average values below 0.4. These regions need to improve both their pure technical efficiency and scale efficiency.

The differences in the DLCEI values among the 12 pilot regions were very small. Basically, almost all of them were greater than or equal to 1, and only Hubei was less than 1. Seven regions’ changes in efficiency (ECH) were lower than their changes in technology (TCH), namely Liaoning, Guizhou, Guangxi, Hunan, Hubei, Shandong, and Zhejiang. The other five regions’ ECH values were larger than their TCH values. The comparison of the ECH and TCH values suggests that these regions need to focus on either technology changes or efficiency improvements to improve their carbon efficiency.

**Figure 6:**
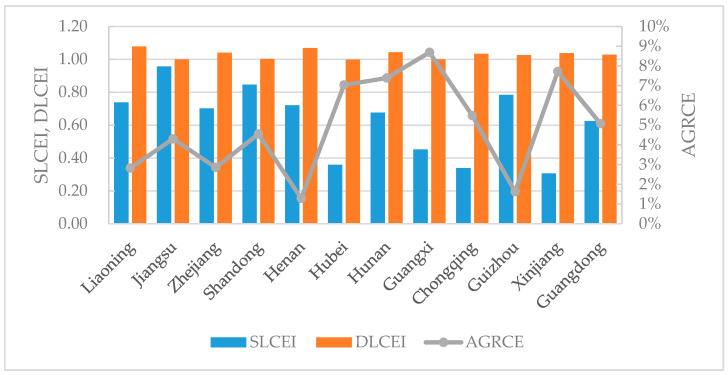
AGRCE, SLCEI, and DLCEI values for the pilot regions. AGRCE: average annual growth rate of the carbon emissions; SLCEI: static carbon emissions efficiency index; DLCEI: dynamic carbon emissions efficiency index.

By plotting the average annual growth rate of the carbon emissions (AGRCE), SLCEI values, and DLCEI values for the twelve pilot regions in a single graph (Figure 6), some insights could be observed. Based on the fluctuations in the AGRCE and SLCEI curves, the 12 regions could be divided into two categories. The first category was the regions with slow growth rates for carbon emissions but high SLCEI values, which were Liaoning, Jiangsu, Zhejiang, Shandong, Henan, and Guizhou. The second category was the regions with high growth rates for carbon emissions but low SLCEI values, namely Guangxi, Xinjiang, Hunan, Hubei, Chongqing, and Guangdong. In particular, Hubei’s DLCEI was 0.9. More attention must be paid to carbon emission management for regions in this second category.

The rankings for SLCEI and DLCEI were basically reversed. For example, Jiangsu Province’s SLCEI value was ranked first, but the DLCEI value was ranked lower. The static carbon emissions efficiency of Jiangsu Province reached a relatively high level, while the room for annual improvement was relatively low. Some regions with lower SLCEI values, such as Henan, Chongqing, and Xinjiang, have a large amount of room for improvement, and thus the DLCEI values were relatively high. 

The existence of large regional differences fully demonstrates that the carbon emissions efficiency of China’s logistics industry still has a large amount of room for improvement. The development of low-carbon green logistics is a complex and systematic process. It not only involves various aspects of the logistics system but also is closely related to the external environment. It is very hard to balance economic, environmental, and social issues. The existing research found that the capital stocks and energy consumption in the logistics industry are the main sources of carbon emissions, which increase the CO_2_ emissions [4,39,40,41,42,43,44,45,46]. The factors that reduce the CO_2_ emissions are adjustments to the energy structure and reductions of the carbon emissions intensity [39,40,41,42,43,44,45,46]. Additionally, the logistics industry is characterized by regional synergy and differentiation, therefore it is necessary to utilize local area advantages and optimize the layout of the logistics industry. The following are policy suggestions for each pilot region to give fully capitalize on regional advantages. 

The central regions such as Henan, Hubei, and Hunan are key transport hubs in China, with a well-connected transport network. It is recommended that local areas should integrate various modes of transport based on their own advantages to form a coordinated system that integrates land, sea, and air transport. Hubei province has very low static and dynamic efficiency, and should actively promote the construction of transport corridors along the Yangtze River. In addition, with the gradual transfer of industries from the eastern to central regions of China, cities such as Zhengzhou in Henan Province and Wuhan in Hubei Province are bound to become logistics centers in the central region of China in the future. The construction of comprehensive and efficient logistics systems can effectively improve the logistics efficiency.

The pilot regions of Guangxi, Chongqing, Guizhou, and Xinjiang belong to the western region. It is recommended to make full use of the advantages of this area, being suitable for agricultural product cultivation and export, and to develop a logistics system that integrates production logistics, urban logistics, and international logistics. Chongqing is located at the intersection of the “Belt and Road” area and the Yangtze River economic belt [16]. It is recommended that a multimodal logistics system should be developed based on the new areas of the two rivers. Guizhou province has proposed to increase its investment in the economic center of Guizhou to further consolidate the position of Guiyang City as the freight hub of the provincial logistics center. Xinjiang, in the context of the “One Belt, One Road” development strategy, is recommended to continue to expand the scale of trade with the five Central Asian countries, optimize its transport routes, and improve its logistics technology.

Liaoning Province is an important transportation hub for the three eastern provinces, which is the old industrial base of northeast China. By accelerating the joint development of the manufacturing and logistics industries in the three eastern provinces, the development and carbon emissions efficiency of the logistics industry can be effectively improved.

Shandong and Guangdong have obvious geographical advantages, and both have excellent coastline and port conditions. It is recommended that Shandong Province use Jinan and Qingdao as centers to focus on developing international trade and shipping logistics and to build a demonstration zone for marine economic development. Guangdong Province can rely on increasing development of cross-strait trade relations, the development of port construction, and becoming a cross-strait logistics relations hub.

### 3.4. Policy Implication

Based on the results and discussion, some policy recommendations from the perspective of China’s strategy for strong transportation are proposed as the following aspects to improve the carbon emissions efficiency of the logistics industry. 

First, we must firmly establish the green low-carbon concept, and optimize the logistics industry energy structure and infrastructure construction. It is recommended to adjust the energy structure by using clean energy, such as tidal energy resources, hydroelectric power generation, and wind power generation. It is necessary to promote the application of new energy logistics vehicles and to promote energy conservation and emissions reduction in freight transport. 

Second, it is suggested to promote the joint development of the economy and the logistics industry. The logistics industry needs to be promoted through increased economic development. It is also necessary to promote the development of the logistics industry and the economy by improving the carbon emissions efficiency of the logistics industry. In the early stage of economic development, the large-scale input of production factors can promote the construction of logistics infrastructure. When economies of scale are limited, structural and technological effects need to be brought into play to promote the development of the logistics industry. Specifically, it is necessary to strengthen the division of labor among regions and promote regional market integration. The development of the logistics industry will be promoted fundamentally by increasing the endogenous growth capacity of the economy.

Third, it is very important to promote the development of smart logistics technology, and improve the efficiency of the logistics industry operations. Regions such as Jiangsu and Zhejiang are approaching the potential best production technologies for carbon emissions efficiency in logistics, and the scope for efficiency improvements in these regions is shrinking. Efforts to further improve the logistics carbon emissions efficiency rely heavily on technological progress. To promote outward migration of logistics productivity under carbon emission constraints, it is necessary to invest heavily in research and innovative energy-saving and emission-reducing technologies. 

Fourth, it is necessary to accelerate the training of professional logistics personnel and improve the logistics industry’s related data statistics. The shortage of professional logistics practitioners has become a bottleneck in the development of the logistics industry. Excellent logistics education should pay attention to the dovetailing of theory and practice inside and outside the education context, and should actively build a training model that combines education and enterprise. In addition, the policies and measures for energy saving and emission reductions in the logistics industry cannot be proposed without reliable data. It is recommended that the government departments improve the collection mechanisms used for logistics-related data at the provincial, municipal, and enterprise levels.

## 4. Conclusions

The static and dynamic carbon emissions efficiency of the logistics industry was evaluated from the perspective of China’s strategy for strong transportation. The analysis of the pilot regions revealed the following conclusions. Compared with the SBM model, the Super-SBM model can further rank the effective DMUs. It was proven to be a more accurate measurement of the carbon emissions efficiency of the logistics industry. Based on the results for the static and dynamic carbon emissions efficiency of the logistics industry, which were obtained by applying the Super-SBM model and the Malmquist index, the pilot regions could be divided into two categories, namely regions with slow growth rates for carbon emissions but high efficiency, and regions with high growth rates for carbon emissions but low efficiency. More attention must be paid to carbon emission management in the second category. Some policy recommendations from the perspective of China’s strategy for strong transportation were proposed to improve the carbon emissions efficiency of the logistics industry. From the new perspective of the strategy for strong transportation, we provided a basis for making decisions for high-efficiency emissions reduction measures, which could encourage the pilot regions to take the lead in achieving the goal of construction a strong transportation network.

This paper also has several limitations. Because data from the logistics industry are not always available, the data from transportation, warehousing, and postal services were used as a substitute, which may have led to inaccurate data. Data on CO_2_ emissions from the logistics industry cannot be obtained directly from the relevant authorities, so we used the consumption of various fossil fuels to estimate the CO_2_ emissions from the logistics industry. Therefore, the data may have caused biased estimations of the results, which is a common weakness of empirical studies. In future studies, the changes of the carbon emissions efficiency of the logistics industry in the pilot regions will be continuously tracked, and the effects of the implementation of China’s strategy for strong transportation will be evaluated through data comparisons over five years or even longer.

## Figures and Tables

**Table 1:** Results for the main fuel coal folding coefficient, calorific value, carbon content, and CO_2_ emissions coefficient.

Energy Type	*NCV* (kJ/kg)	*CEF* (kg/GJ)	*COF* (kg/TJ)	CO_2_ Emissions Factor (kgCO_2_/kg)
Raw coal	20,908	26.37	0.94	1.9003
Petrol	43,070	18.9	0.98	2.9251
Kerosene	43,070	19.5	0.98	3.0179
Diesel	42,652	20.2	0.98	3.0959
Fuel oil	41,816	21.1	0.98	3.1705
Coke	28,435	29.5	0.93	2.8604
Liquefied petrol	50,179	17.2	0.98	3.1013

*NCV*: average low calorific value; *CEF*: carbon emissions coefficient; *COF*: carbon oxidation factor.

**Table 2:** A description of the data sources for each indicator.

Indicator	Description	Data Source	Units
Employment	Number of persons actually employed at the end of each year in transportation, warehousing, and postal services	China City Statistical Yearbook (2013–2017)	10,000 people
Capital stock	The fixed capital stock for transportation, warehousing, and postal services	China City Statistical Yearbook (2013–2017)	1 billion yuan
Energy consumption	The energy consumption for transportation, warehousing, and postal services	China Energy Statistical Yearbook (2013–2017)	10,000 tons of standard coal
Infrastructure	Sum of railway mileage, road mileage, and inland waterway mileage	China City Statistical Yearbook (2013–2017)	10,000 km
Production value	Production value of transportation, warehousing, and postal services	China City Statistical Yearbook (2013–2017)	1 billion yuan
CO_2_ emissions	Estimated by IPCC, which was introduced in Section 2.1.	China Energy Statistical Yearbook (2013–2017)	10,000 tons

**Table 3:** Values for the static carbon emissions efficiency index of the logistics industry (SLCEI) for the pilot regions under SBM and Super-SBM models.

Province	2013		2014		2015		2016		2017		Mean	Rank
	SBM	Super SBM	SBM	Super SBM	SBM	Super SBM	SBM	Super SBM	SBM	Super SBM	Super SBM	Super SBM
Liaoning	0.553	0.553	0.555	0.555	0.759	0.759	0.825	0.825	**1.000**	1.049	0.748	5
Jiangsu	**1.000**	1.048	0.925	0.925	0.913	0.913	0.947	0.947	**1.000**	1.050	0.977	1
Zhejiang	0.666	0.666	0.676	0.676	0.666	0.666	0.727	0.727	0.774	0.774	0.702	6
Shandong	**1.000**	1.023	0.729	0.729	0.727	0.727	0.776	0.776	**1.000**	1.002	0.851	2
Henan	0.550	0.550	0.723	0.723	0.674	0.674	0.796	0.796	0.857	1.049	0.758	4
Hubei	0.354	0.354	0.371	0.371	0.371	0.371	0.337	0.337	0.364	0.364	0.359	10
Hunan	0.587	0.587	0.624	0.624	0.575	0.575	0.590	0.590	**1.000**	1.006	0.676	7
Guangxi	0.469	0.469	0.426	0.426	0.444	0.444	0.450	0.450	0.474	0.474	0.453	9
Chongqing	0.321	0.321	0.354	0.354	0.320	0.320	0.335	0.335	0.365	0.365	0.339	11
Guizhou	0.662	0.662	0.705	0.705	0.771	0.771	0.782	0.782	**1.000**	1.086	0.801	3
Xinjiang	0.282	0.282	0.303	0.303	0.297	0.297	0.338	0.338	0.312	0.312	0.306	12
Guangdong	0.564	0.564	0.603	0.603	0.614	0.614	0.646	0.646	0.701	0.701	0.626	8

SBM: slack-based measurement.

**Table 4:** Results for the dynamic carbon emissions efficiency index of the logistics industry (DLCEI) for the pilot regions.

	2013–2014	2014–2015	2015–2016	2016–2017	Mean	Rank
Liaoning	0.986	1.311	0.943	1.071	1.078	1
Jiangsu	0.965	0.992	1.021	1.023	1.000	10
Zhejiang	1.015	1.011	1.079	1.054	1.040	4
Shandong	0.875	1.009	1.041	1.088	1.003	9
Henan	1.236	0.942	1.08	1.018	1.069	2
Hubei	1.032	1.027	0.858	1.077	0.999	12
Hunan	1.138	0.925	1.029	1.079	1.043	3
Guangxi	0.854	1.046	1.024	1.074	1.000	11
Chongqing	1.14	0.903	1.038	1.051	1.033	6
Guizhou	0.984	1.042	1.000	1.073	1.025	8
Xinjiang	1.043	1.017	1.184	0.907	1.038	5
Guangdong	1.008	0.989	1.089	1.026	1.028	7
